# Correction: A high affinity pan-PI3K binding module supports selective targeted protein degradation of PI3Kα

**DOI:** 10.1039/d4sc90011a

**Published:** 2024-01-15

**Authors:** Werner Theodor Jauslin, Matthias Schild, Thorsten Schaefer, Chiara Borsari, Clara Orbegozo, Lukas Bissegger, Saule Zhanybekova, Danilo Ritz, Alexander Schmidt, Matthias Wymann, Dennis Gillingham

**Affiliations:** a Department of Chemistry, University of Basel 4056 Basel Switzerland dennis.gillingham@unibas.ch; b Department of Biomedicine, University of Basel 4031 Basel Switzerland matthias.wymann@unibas.ch; c Proteomics Core Facility, Biozentrum University of Basel 4056 Basel Switzerland

## Abstract

Correction for ‘A high affinity pan-PI3K binding module supports selective targeted protein degradation of PI3Kα’ by Werner Theodor Jauslin *et al.*, *Chem. Sci.*, 2024, https://doi.org/10.1039/D3SC04629J.

The authors regret that in the published article, descriptions of the contributions made by two of the authors were left out of the Author contributions statement. The corrected version of the Author contributions statement can be found below:

W. J., M. S., M. W., and D. G. collaborated in all aspects of project design and conception. W. J. performed chemical synthesis and initial cellular testing of all molecules, including mechanistic experiments. W. J. and M. S. designed the proteomics experiments and performed the initial experimental setup in collaboration with D. R. and A. S., D. R. and M. S. performed the global and targeted proteomics in collaboration under the supervision of D. G. and A. S. Cellular engineering was done by S. Z. and testing of engineered cells was done by W. J. Measurement of binding data and In-Cell Western assays were performed by L. B. and C. O. under the supervision of M. W. C. B. helped with the initial designs of PROTAC structures and contributed to the chemical synthesis of initial VHL targeting molecules and T. S. performed the cellular studies examining degradation levels across several mutated cell lines. The first draft of the manuscript was prepared by W. J., M. S., M. W. and D. G. Refinement was done with contributions from all authors.

The Royal Society of Chemistry apologises for these errors and any consequent inconvenience to authors and readers.

## Supplementary Material

